# RIPK3 signaling and its role in regulated cell death and diseases

**DOI:** 10.1038/s41420-024-01957-w

**Published:** 2024-04-29

**Authors:** Yaqi Zhou, Yaxuan Xiang, Sijie Liu, Chenyao Li, Jiaheng Dong, Xiangrui Kong, Xinying Ji, Xiaoxia Cheng, Lei Zhang

**Affiliations:** 1 School of Basic Medical Sciences, Henan University, Kaifeng, 475004 China; 2 Department of Pathology, the Second People’s Hospital of Jiaozuo; The First Affiliated Hospital of Henan Polytechnic University, Jiaozuo, 454000 China; 3 Faculty of Basic Medical Subjects, Shu-Qing Medical College of Zhengzhou, No. 6 Gong-Ming Rd, Mazhai Town, Erqi District, Zhengzhou, Henan 450064 China; 4 Wushu College, Henan University, Kaifeng, 475004 China

**Keywords:** Cancer therapy, Necroptosis

## Abstract

Receptor-interacting protein kinase 3 (RIPK3), a member of the receptor-interacting protein kinase (RIPK) family with serine/threonine protein kinase activity, interacts with RIPK1 to generate necrosomes, which trigger caspase-independent programmed necrosis. As a vital component of necrosomes, RIPK3 plays an indispensable role in necroptosis, which is crucial for human life and health. In addition, RIPK3 participates in the pathological process of several infections, aseptic inflammatory diseases, and tumors (including tumor-promoting and -suppressive activities) by regulating autophagy, cell proliferation, and the metabolism and production of chemokines/cytokines. This review summarizes the recent research progress of the regulators of the RIPK3 signaling pathway and discusses the potential role of RIPK3/necroptosis in the aetiopathogenesis of various diseases. An in-depth understanding of the mechanisms and functions of RIPK3 may facilitate the development of novel therapeutic strategies.

## Facts

- The RIPK3 protein plays an indispensable role in necroptosis.
- RIPK3 is involved in the pathological process of several infections, aseptic inflammatory diseases, and tumors.
- RIPK3 is involved in the release of inflammatory factors; however, the mechanisms through which RIPK3 promotes inflammatory responses warrant further investigation.

## Open questions

- Can RIPK3 be used as a drug target for the treatment of cancer?
- Is RIPK3 cross-functional between necroptosis and other forms of regulated cell death?
- Can clinically applicable combination therapies be developed to alleviate necroptosis and necrosis-driven inflammation?

## Introduction

Necrosis has been traditionally considered a passive form of cell death that cannot be regulated [1]. However, extensive research in the field of cell death has revealed that necrosis can be regulated in a programmed manner through a signaling pathway called necroptosis [2, 3]. Receptor-interacting protein kinases (RIPKs) are a family of serine/threonine kinases [4], with the first member, RIPK1, being identified in 1995 [5]. RIPK1 and RIPK3 play an important role in necroptosis. The involvement of RIPK1 in necroptosis was discovered in the early 2000s [6], whereas that of RIPK3 was discovered in 2009. During necroptosis, RIPK3 interacts with RIPK1 and serves as an essential downstream factor of RIPK1. At present, RIPK3 is an important focus of research in the field of necroptosis [7–10].

Stimulating the activation of Mixed lineage kinase domain-like pseudokinase (MLKL) and triggering necroptosis are classical functions of RIPK3. However, recent studies have shown that RIPK3 performs various other functions in cells and may play diverse roles in necroptosis. The non-classical functions of RIPK3 include the induction of inflammasome activation and cytokine production, stimulation of ROS production, and regulation of autophagy and cell proliferation. RIPK3-regulated necroptosis is involved in the pathological process of systemic inflammatory diseases, ischemia–reperfusion injury, and neurodegeneration. The RIPK3 signaling pathway is associated with the pathogenesis of various cancers and exhibits both tumor-promoting and -suppressive activities, indicating that RIPK3 plays diverse roles in tumor development, metastasis, and recurrence. This review summarizes recent studies on the mechanisms of the RIPK3 signaling pathway, discusses the key role of RIPK3 in disease treatment and tumorigenesis, and highlights its potential applicability as a new drug target.

## Structural features of RIPK3

The RIPK family is composed of seven protein kinases with serine/threonine kinase activity. As a sensor of both intracellular and extracellular events, RIPKs contribute to immunological responses, inflammation, and cell death. Each RIPK family member is involved in different processes based on its function [11]. Structurally, all RIPK family members have a serine/threonine kinase domain at the N-terminus. The highly homologous kinase structure suggests that RIPK family members have some common biological functions. However, the C-terminus of RIPKs is different and can specifically bind to a corresponding protein to exert different biological effects. The C-terminal region of RIPK1 contains a death domain (DD), that of RIPK2 contains a caspase recruitment domain (CARD) that is unique to aspartate, and that of RIPK4 contains ankyrin (ANK) repeats. These unique C-terminal structures suggest varying mechanisms of signal recruitment and transmission. Although RIPK3 lacks a unique C-terminal structure, it shares a receptor-interacting protein homotypic interaction motif (RHIM) with RIPK1. RHIM, a structure that is absent in other members of the RIPK family, enables the interaction between RIPK3 and RIPK1, which is essential for necroptosis.

The human RIPK3 gene is located on chromosome 11, spanning 10 exons and approximately 40 kb of genome-wide DNA. The RIPK3 protein comprises 518 amino acids and has a molecular weight of approximately 50 kDa. The kinase domain and RHIM are necessary for the normal functioning of RIPK3. RHIM allows RIPK3 to interact with other RHIM-containing proteins, such as RIPK1, ZBP1, and TRIF [12].

## Role of RIPK3 in necroptosis

### Necroptosis

The life of multicellular organisms is closely associated with cell death. Cell death is classified as regulated cell death (RCD) or accidental cell death (ACD). ACD is an uncontrolled process that occurs in response to accidental injury. It is a sign of pressure, injury, or infection and is associated with tissue damage and etiological factors. On the contrary, RCD is mediated by well-structured interconnected signaling cascades and molecular mechanisms and hence can be managed by genetic factors and pharmacological agents. The term “programmed cell death” refers to RCD that occurs under physiological conditions, such as the development of embryos to maintain homeostasis in adult tissues, whereas RCD is triggered by exogenous disturbances in the intracellular or extracellular microenvironment and occurs after the failure of adaptive processes such as autophagy or the endoplasmic reticulum (ER) stress response. Initially, apoptosis was considered the only type of controlled cell death, whereas necrosis was considered an uncontrolled type of ACD [13]. However, with extensive research on the types of cell death and the mechanism The Cell Death Nomenclature Committee has revised the definition of RCD based on molecular mechanisms, genetic characteristics, immunomodulatory modes, and morphological characteristics. Necroptosis, pyroptosis, ferroptosis, and cuproptosis are other unique types of RCD mechanisms [14–17] (Table 1). Apoptosis refers to cell death induced by cysteine proteases. Upon activation of cysteine proteases, the hydrolysis cascade enhances the activity of the apoptosis pathway, leading to irreversible cell death. After apoptosis is initiated, the cells undergo DNA fragmentation, plasma membrane blebbing, cytoplasmic and nuclear condensation, apoptotic vesicle formation, and apoptotic body clearance via phagocytosis. Necrosis is a non-energetic process that results from inadvertent responses to various stress stimuli. It is characterized by cell swelling, plasma membrane rupture, and cell content leakage, resulting in inflammation and immune-stimulating reactions. Necroptosis, commonly referred to as “programmed necrosis,” depends primarily on RIPK1, RIPK3, and MLKL and does not require caspases. Similar to apoptosis, programmed necrosis requires energy and specific proteins; therefore, kinase activity is essential for apoptosis. Although necroptosis and apoptosis share the same upstream molecular mechanisms, their outcomes are different. Similar to necrosis, programmed necrosis leads to cell swelling, plasma membrane disruption, cell leakage, and activation of the immune system, with strong proinflammatory effects [18]. However, the mechanism of immune activation differs between necroptosis and classical necrosis. Necroptosis plays diverse roles in innate immunity and leads to the release of endogenous danger signals called DAMPs [19, 20]. Innate immune cells respond to pathogens and potentially dangerous cells (such as infected or tumorigenic cells) after recognizing DAMPs through pattern recognition receptors (PRRs). In addition, the immunological response triggered by necroptosis is not limited to local tissues but may expand and induce a systemic immune response.

**Table 1:** Characteristics of the main RCD types.

Type	Morphological features	Biochemical features	Major regulators	Major inhibitors (target)
Apoptosis	Cell shrinkage, condensation of chromatin, blebbing of membranes, and formation of apoptotic bodies	DNA breakage, triggering of caspases, phosphatidylserine exposure, mitochondrial malfunction, release of cytochrome c, and altered expression and activation of Bcl-2 family proteins	Caspase (caspase-2, caspase-3, caspase-7, caspase-8, caspase-9, and caspase-10) BCL2 family (BAK1, BAX, BOK, BID, BCL2L1, MCL1, BCL2L2, and BCL2L10), TP53, IAP1/2, and XIAP	Z-VDVAD-FMK (caspase-2), Z-VAD-FMK (pan-caspase), Z-DEVD-FMK (caspase-3, caspase-7, and caspase-10), and Z-IETD-FMK (caspase-8)
Necroptosis	Cell swelling and dissolution, appearance of pores on the cell membrane, and plasma membrane rupture	Caspase-independent phosphorylation of MLKL by RIPK1/RIPK3, release of DAMPs, and necrosome assembly	RIPK1, RIPK3, MLKL, caspase-8, cIAPs, and LUBAC	Nec-1s, Nec-1 RIPA-56 (RIPK1), GSK840, GS843, GSK872 (RIPK3), necrosulfonamide, and TC13172 (MLKL)
Pyroptosis	Cell swelling and lysis, inflammasome activation, membrane rupture, plasma membrane blebbing, and mild chromatin condensation	Caspase-dependent process; activation of caspase-1, caspase-3, and GSDMD; GSDMD cleavage; and release of Ll-18 and IL-1β	Caspase-1, caspase-4, caspase-5, caspase-11, caspase-7, GSDMD, PKA, and GPX4	Z-YVAD (OMe)-FMK, VX765 (caspase-1), Ac-FLTD-CMK (GSDMD cleavage), and MCC950 (NLRP3 inflammasome)
Autophagy	Autophagic vacuolization	Caspase-independent process, lysosomal activity, LC3 lipidation, autophagosome formation, and increased autophagic flux	AMPK, ULK, VPS34, and mTOR	HCQ/CQ (lysosome)
Ferroptosis	Cell swelling, appearance of pores on the cell membrane, decreased mitochondrial volume, rupture of the outer membrane of mitochondria, decreased size or absence of the mitochondrial crest, and increased mitochondrial membrane density	Caspase-independent iron accumulation, membrane lipid oxidation and peroxidation dependent on Fe2 + , and suppression of the Xc-system/GSH/GPX4 pathway	Fe2 + , TFRC, NCOA4, ACSL4, CARS, LPCAT3, VDAC2/3, ALOX15, GLS2, DPP4, BAP1, BECN1, PEBP1, RAB7A, ALK4/5, TP53, SLC7A11, GPX4, NFE2L2, and OTUB1	Deferoxamine, deferiprone (Fe); sorafenib sulfasalazine, erastin, glutamate (system Xc-); ferrostatin-1, liproxstatin-1, vitamin E, NAC (ROS); vildagliptin, alogliptin (DPP4); selenium (GPX4)
Cuproptosis	Smaller mitochondria and increased rupture of the mitochondrial membrane	Protein aggregation and proteotoxic stress	Positive: FDX1, DLAT, LIAS, CTR1, RTK, ATK, ERK, and PI3K Negative: ATP7A, ATP7B, MT, GSH, ATOX1, CCS, and SOD1	TTM (Cu)

In the necroptosis pathway, RIPK3 is an important signaling molecule located downstream of RIPK1. Studies have shown that necroptosis occurs when the following two conditions are met in vitro: expression of RIPK3 in cells and inhibition of caspase-8 activity by drugs or virus-derived caspase inhibitors. Therefore, RIPK3 is an important molecular switch that determines the initiation of necroptosis or apoptosis in cells.

### Upstream signals that stimulate RIPK3 activation in necroptosis

**Fig. 1:**
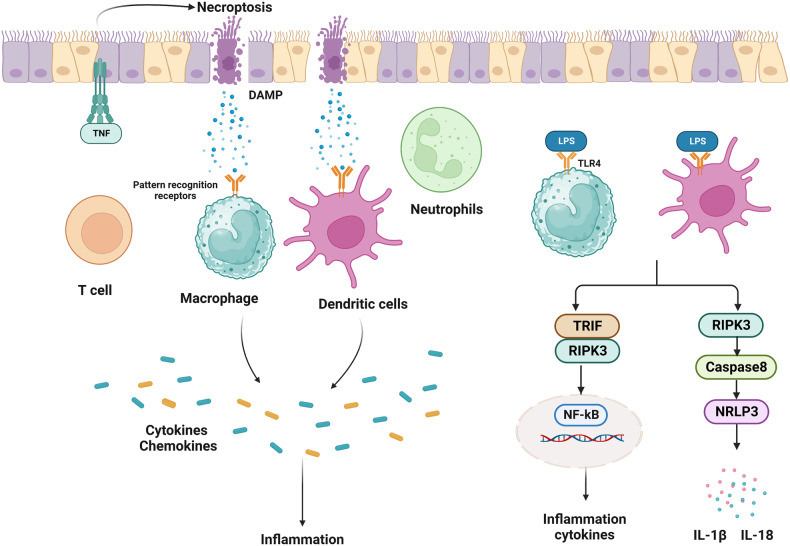
RIPK3 signaling-mediated necroptosis. Multiple stimuli activate RIPK3 to mediate necroptosis. RIPK3 can be activated by various receptors other than TNF receptors, including FAS, TRAIL, TLR3/4, and ZBP1, when bound by their corresponding ligands.

In response to various cellular stress stimuli, RIPK3 is activated by suitable ligands via corresponding receptors, including death receptor ligands such as TNF, FASLG, and TRAIL. FAS was the first receptor discovered to induce RIPK1-dependent necroptosis. FAS/TRAIL and TNF-R1 induce necroptosis through a similar mechanism, which involves the assembly of the DISC on the plasma membrane and RIPK1-mediated induction of necrosome formation and RIPK3 activation [21]. When caspase-8 is suppressed, depletion of cIAP facilitates the interaction between RIPK1 and FAS/TRAIL and enhances the formation of intracytoplasmic ribosomal complexes, thereby inducing necroptosis (Fig. 1). The RIPK3 signaling pathway has been primarily investigated in the context of TNF-α stimulation. TNF is a multifunctional cytokine that remarkably aggravates inflammation, cell death, and tissue destruction. TNFR1, FAS (also known as CD95), DR3, TRAILR1 (also known as DR4), TRAILR2 (also known as DR5), and DR6 are six human DRs belonging to the TNF receptor superfamily. The TNFR1 signaling pathway has been investigated most extensively [22]. Activation of TNFR1 by TNF leads to the formation of a homotrimer. When TNF binds to TNFR1 on the plasma membrane, TRADD, cIAP1/2, RIPK1, and TRAF2/5 are immediately recruited to the plasma membrane to create complex I [12, 23]. The death domain of TNFR1 interacts with the death domains of both RIPK1 and TRADD. Upon its recruitment to complex I, RIPK1 undergoes polyubiquitination and phosphorylation. Ubiquitination of the RIPK1 protein is primarily mediated by the c-IAP1/2 and LUBAC proteins. cIAP1, cIAP2, and cIAP1/2 with E3 ubiquitin ligase activity are recruited to this complex via ubiquitination of TRADD at K63, consequently enabling polyubiquitination of RIPK1. In particular, cIAP1/2 adds a K63-linked polyubiquitin chain to the K376 residue of RIPK1 [24–27]. TAK1 and TAB interact with polyubiquitinated RIPK1, activating the NF-κB signaling pathway. The K63-linked polyubiquitin chain in complex I is recruited by LUBAC, which comprises SHARPIN, HOIP, and HOIL1 [28]. LUBAC induces the formation of an M1-linked ubiquitin chain on RIPK1, thereby promoting the recruitment of NEMO to complex I. IKKα (also known as IKK1) and IKKβ (also known as IKK2) form the IKK complex, with NEMO being one of the regulatory subunits. Direct phosphorylation of IKKβ by activated TAK1 activates the IKK complex which in turn phosphorylates IκBα (an antagonistic regulator of NF-κB) and activates the NF-κB signaling pathway. In addition, recruitment of the IKK complex to complex I activates ERK, JNK, and p38 and promotes the expression of inflammatory genes [29]. The NF-ΚB and JNK signaling pathways regulate various pro-survival genes, such as cIAPs, BCL-Xl, and cFLIP, with c-IAP1/2 and cFLIP serving as anti-apoptotic genes [30, 31]. Although cFLIP is a homodimer of caspase-8 and lacks caspase function, it can bind to caspase-8 and prevent its activation, consequently protecting cells from caspase-8-mediated apoptosis. In addition, cIAP2 promotes the ubiquitin-mediated degradation of IκB, and activates NF-κB and enhances cell survival by releasing NF-κB into the nucleus. Therefore, complex I primarily mediates TNF-α-stimulated survival and proinflammatory signaling. TNF-α-induced activation of RIPK3 signaling requires the dissociation of complex I, which is mediated by the ubiquitination enzymes A20 [32], CYLD [33], and OTULIN [34]. These enzymes remove K63-linked ubiquitin chains from RIPK1. The dissociation of TRADD and RIPK1 from TNFR1 leads to the formation of complex II, which comprises subcomplexes IIa and IIb and complex IIc (a necrosome). After complex I is dissociated, TRADD recruits FADD, which in turn recruits and activates caspase-8 to produce complex IIa, leading to RIPK1-independent apoptosis (RIA) [23, 35]. RIPK1 dissociates from complex I and creates complex IIb under some conditions, such as when cIAP1/2 is deleted, cIAP1/2 is degraded, or IAP inhibitors are present [36]. Complex IIb, which comprises FADD, RIPK1, and FLIPL/caspase-8 heterodimer, promotes RIPK1-dependent apoptosis (RDA), a type of cell death mechanism that requires both RIPK1 and caspase-8 [13]. Therefore, although the presence of RIPK1 can trigger apoptosis, RIPK1 is not essential for apoptosis. When Z-VAD inhibits caspase-8, RIPK1 interacts with RIPK3 through RHIM to create complex IIc (a necrosome). Under physiological conditions, the heterodimer formed by caspase-8 and cFLIPL promotes the production of caspase-8 oligomers to induce apoptosis. However, the activity of necrosomes is negatively regulated by pro-caspase-8/cFLIPL heterodimers. These heterodimers are cleaved by RIPK1 (Asp325 in mice and Asp324 in humans) and RIPK3 (Asp333 in mice and Asp328 in humans), resulting in the inhibition of necroptosis [37–39]. cFLIPS competes with cFLIPL to form a heterodimer with the caspase-8 precursor. The heterodimer formed by pro-caspase8 and cFLIPS inhibits apoptosis and promotes the formation of necrosomes and necroptosis. Consequently, the cFLIP isoform in complex II controls whether cell death occurs through caspase-dependent apoptosis or necroptosis, which is regulated by RIPK3. In necrosomes, activated RIPK1 can bind to RIPK3 through RHIM. Upon the binding of RIPK1 with RIPK3, additional RIPK3 is recruited to the complex, activating RIPK3 phosphorylation, which is a crucial step in the induction of necroptosis. Although RIPK1 does not directly phosphorylate RIPK3, it is required for TNF-induced RIPK3 activation and necroptosis. In the cytoplasm, RIPK1 is activated via autophosphorylation at residues Ser14/15, Ser20, Ser161, and Ser166 (Ser14/15, Ser161, Ser166, and Thr169 in mouse) [7, 40, 41]. Autophosphorylation of RIPK1 at Ser166 is crucial for RIPK1-mediated apoptosis and necroptosis [42, 43]. Activated RIPK1 activates RIPK3, leading to the autophosphorylation of RIPK3 (at T231 and S232 in mice and S227 in humans) [7, 9]. A recent study showed that TRADD, a death domain adapter protein, can directly bind to RIPK3 to enhance TNF-induced necroptosis in cells lacking RIPK1 [44].

PRRs, including Toll-like receptors (TLR3 and TLR4), TRAILR and ZBP1, as well as TNFR1-mediated conventional necroptosis result in the formation of necrosomes and activation of RIPK3. The receptors bind to RIPK3 through RHIM in each of these three pathways [45]. TLR4 and TLR3 can be activated by LPS and dsRNA, respectively. ZBP1, a cytoplasmic nucleic acid sensor, is activated by intrinsic DNA and RNA produced upon DNA damage-inducing treatments, such as radiation therapy and chemotherapy, and viral infection. The adaptor protein of TLR3 is TRIF, whereas the two main adaptor proteins of TLR4 are TRIF and MYD88 [46]. Similar to RIPK1, RIPK3 interacts with TRIF and ZBP1 through RHIM to activate the RIPK3 signaling pathway. TLR signaling activates RIPK3 and induces necroptosis, leading to the death of infected cells to protect the host during viral or microbial infection. Necroptosis directly triggered by the TRIF/RIPK3/MLKL pathway can be blocked by caspase-8. Downregulation of RIPK1 enhances both TRIF- and ZBP1-mediated activation of RIPK3, indicating that RIPK1 competes with these two factors to bind to RIPK3. During HSV-1 infection, some viruses produce RHIM-containing proteins, such as ICP6, which directly activate RIPK3 and cause necroptosis in infected cells. ICP6 selectively promotes necroptosis in mice but is inactive in humans [47, 48].

Necroptosis can be induced by other PRRs, such as DDX58 (also known as RIG-I) and INFAR1 (an interferon receptor). In the presence of z-VAD-FMK, activation of JAK1 through the cognate receptor IFNAR1 results in the formation of the IRF9–STAT complex, which eventually causes necroptosis. With the assistance of interferon regulatory factor 1 (IRF1), IFN may trigger necroptosis in cancer cells when combined with Smac mimics.

### Downstream signals that activate RIPK3 in necroptosis

The primary functions of RIPK3 signaling include the induction of necroptosis and activation of MLKL. In the necrosis complex, RIPK1 phosphorylates RIPK3, and activated RIPK3 in turn phosphorylates MLKL at T357 and S358. Upon phosphorylation, MLKL forms a trimer and exposes its four-helix bundle domain. After MLKL localizes to the plasma membrane, its conformation changes, increasing its plasma membrane-binding ability. MLKL can bind to phosphatidylinositol phosphate (PIP) and cardiolipin (CL) in the plasma membrane. Studies have shown that oligomerized MLKL can interact with transient receptor potential melastatin-related 7 (TRPM7) to mediate extracellular Ca^2+^ influx, leading to plasma membrane damage [49]. In addition, the MLKL complex on the cytoplasmic membrane can increase the influx of sodium ions, either independently or through other membrane proteins, thereby increasing the intracellular osmotic pressure and eventually leading to cell swelling and plasma membrane disruption. Phosphorylation of MLKL, Ca^2+^ influx, and translocation of phosphatidylserine (PS) to the surface of the cell membrane, which leads to membrane damage, can activate the endosomal sorting complex required for transport III (ESCRT-III). ESCRT-III controls the duration of plasma membrane integrity and ensures cell survival [50, 51]. Phosphoglycerate mutase family member 5 (PAGM5) has been identified as a target of necrosomes. Phosphorylated MLKL recruits and phosphorylates PGAM5 through RIPK3. PGAM5 is a mitochondrial protein that exists in two forms, PGAM5l and PGAM5s. PGAM5s recruits mitochondrial division factor kinetic-related protein 1 (Drp1), which is activated upon dephosphorylation. Activated Drp1 triggers mitochondrial cleavage and ROS production, eventually inducing necroptosis [52]. In addition to the RIPK1–RIPK3–MLKL signaling cascade, activation of CaMKII is another mechanism of necroptosis induction. Activated CaMKII stimulates the opening of procyclin D (CypD)-mediated MPTP, resulting in dysfunctional mitochondrial membrane potential depolarization and necroptosis of cardiomyocytes. Studies have validated that the heart can be targeted to protect against ischemia- and oxidative stress-induced necroptosis and myocardial remodeling via the RIPK3–CaMKII–MPTP pathway [53]. ROS, ANT, and RNS play an important regulatory role in programmed necrosis. RIPK3 promotes ROS production by activating glycogen phosphorylase (PYGL), glutamate ammonia ligase (GLUL), and glutamate dehydrogenase (GLUD1).

Excessive accumulation of ROS activates the pro-necrotizing function of JNK, thus preventing TNF-α-induced apoptosis and inducing necrosis or necroptosis. The exchange of ADP for ATP by the mitochondrial enzyme ANT is necessary for the production of mitochondrial ATP. Blockade of the ANT-mediated transport of cytoplasmic ADP results in a decreased amount of mitochondrial ATP, leading to the activation of necroptosis. A study found that excess nitric oxide triggers RNS production, which is crucial for the oxidation and peroxidation of lipids and proteins. Furthermore, nitration can trigger RIPK1 and RIPK3-dependent necroptosis. In particular, nitration of nDUFB8 present in mitochondrial supercomplexes can lead to dissociation of the supercomplexes and depolarization of mitochondria, both of which are associated with necroptosis.

## Non-necrotic function of RIPK3

### Function of RIPK3 in NF-κB signaling

Nuclear factor-κB (NF-κB) is a family of proteins that play a key role in the expression of pro-inflammatory and pro-survival factors [54]. It comprises five proteins, namely, RelA (p65), RelB, c-Rel, NF-κB1(p50), and NF-κB2(p52). These proteins have a highly conserved Rel homology region (RHR) at the N-terminus. The N-terminal domain (NTD) and C-terminal domain (CTD) collectively constitute the RHR. The nuclear localization sequence (NLS) present in the CTD can bind to DNA and induce dimerization as well as nuclear translocation [55]. For necrotic apoptosis, the main regulatory mechanism of RIPK3 in NF-κB signaling is that RIPK3 contains the RHIM and is controlled by other RHIM-containing proteins [56]. Such as RIPK1 activation, RIPK3 through RHIM self-assembly, RHIM phosphorylation and activate the substrate MLKL structure, lead to the NF-κB activation, membrane rupture [57], and programmed necrosis (necroptosis) [58]. Some degree of inflammation may result from the release of DAMPs after membrane rupture [59]. In non-necroptosis, RIPK3 regulates NF-κB mainly through the TRIF–RIPK1 and ZBP1–RIPK1 signaling pathways. In the TRIF–RIPK1 signaling pathway [60], RIPK3 competes with RIPK1 to bind to Toll-like receptor adaptor molecule (TRIF) [61] and blocks the signal transduction of NF-κB through TRIF–RIPK1 signaling. In the ZBP1–RIPK1 signaling pathway, RIPK3 can cooperate with ZBP1–RIPK1 signaling to NF-κB, promoting the activation of NF-κB by ZBP1 [62]. Therefore, RIPK3 either activates or inhibits the activation of NF-κB through different mechanisms.

The effects of RIPK3 on NF-κB vary depending on the cell state. When the cells are in a resting state, IκB-α (an inhibitor of NF-κB) and two subunits of NF-κB, namely, p65 and p50, are present in the cytoplasm in an inactivated state. Mediated by MyD88 TLR4/NF-κB signaling pathways activated IKK.The activated IKK ubiquitinates, phosphorylates, and degrades IκB-α, leading to the activation of both NF-κB subunits from an inactive state and their translocation from the cytoplasm to the nucleus (especially the p65 subunit). The activated subunits bind to the corresponding pro-inflammatory genes, thereby initiating their transcription and inducing inflammation. TNF and TLR ligands can induce the phosphorylation and degradation of IκB-α in RIPK3-deficient mouse embryonic fibroblasts and bone marrow-derived macrophages (BMDMs) [63, 64]. Degradation of IκB-α stimulates the release of NF-κB from the cytoplasmic NF-κB/IκBα complex. The released NF-κB is activated to form dimers and rapidly undergoes nuclear translocation. RIPK3 is not involved in the formation of NF-κB dimers, indicating that it does not affect the activation of NF-κB. On the contrary, RIPK3 plays an important regulatory role in TLR4-induced NF-κB activation and cytokine production in bone marrow-derived dendritic cells (BMDCs) producing granulocyte–macrophage colony-stimulating factor (GM-CSF) and interleukin-4 (IL-4). In DCs, RelB binds to p50, instead of the conventional NF-κB chaperone p52, and regulates TLR-mediated cytokine expression [65]. The phosphorylation and degradation of IκBα occur normally but the nuclear translocation of RelB-p50 heterodimers is severely impaired in RIPK3-expressing BMDCs stimulated with TLR4. Therefore, RIPK3 not only promotes the activation of NF-κB through the TLR4 pathway but also affects its activation by interfering with the nuclear translocation of NF-κB dimers.

### RIPK3 induces inflammasome activation and cytokine production

**Fig. 2:**
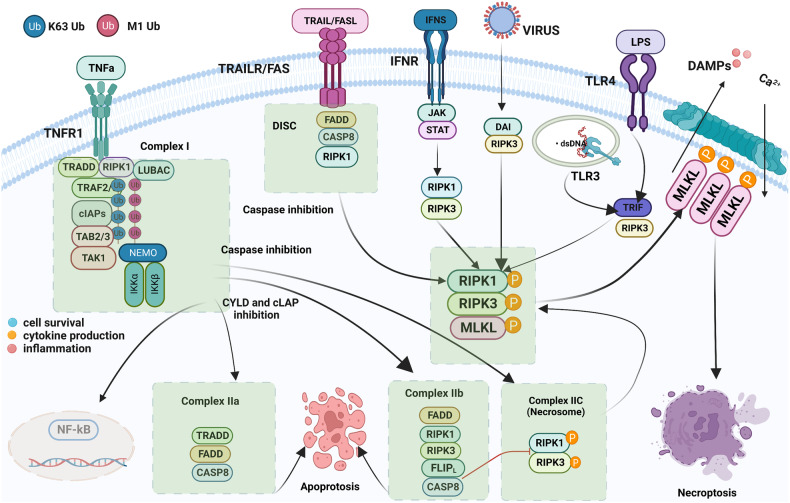
RIPK3 induces inflammasome activation and cytokine production. Cells undergoing RIPK3-induced necroptosis release intracellular DAMPs. In dendritic cells (DCs) and macrophages, RIPK3 regulates the production of cytokines/chemokines by regulating the NF-κB pathway. The RIPK3 protein induces the production of IL-1β and IL-18 through the caspase-8–RIPK1–NLRP3 axis. In addition, it induces the production of NF-kB through the TRIF–RIPK3 pathway to stimulate the production of cytokines.

Cells undergoing RIPK3-induced programmed necrosis release intracellular DAMPs [66]. DAMPs mainly include IL-1 family cytokines, mtDNA, shock proteins, and the S100A8 protein. Recognition of DAMPs by PRRs induces the secretion of various cytokines and chemokines to activate the immune response and trigger inflammation. However, recent studies have shown that RIPK3 can induce inflammation by producing cytokines/chemokines in a cell death-independent manner (Fig. 2). For example, in dendritic cells (DCs) and macrophages, the production of cytokines/chemokines is regulated through the NF-κB pathway and the transcription of cytokine-/chemokine-coding genes [67]. In addition, RIPK3 promotes the activation of the Nod-like receptor family pyrin domain-containing 3 (NLRP3) inflammasome. Inflammasomes are a complex of various proteins produced in bone marrow cells and serve as an important component of the innate immune system. In response to external pathogens or injuries, signals are transmitted to the immune system to initiate inflammation. To date, various inflammasomes have been identified, including NLRP1, NLRP3, NLRC4, and AIM2 inflammasomes [66]. NLRP3 is a member of the nucleotide-binding domain and leucine-rich repeat (NLR) family of intracellular PRRs [68]. RIPK3 primarily activates the NLRP3 inflammasome. Further processing and maturation of cytokines such as IL-1β and IL-18 [12]. RIPK3 promotes the activation of inflammatory corpuscles through two distinct mechanisms related to the activity of caspases [12]. First, in cells expressing caspase-8, the RIPK3 protein mediates the activation of inflammasomes through the caspase-8–RIPK1–NLRP3 axis. This mechanism does not require the kinase activity of RIPK3 [69]. Second, in cells lacking caspase-8, RIPK3 mediates the phosphorylation of MLKL through its kinase activity. Phosphorylated MLKL can directly induce potassium efflux and activate the NLRP3 inflammasome [70, 71]. A study showed that RIPK3 promoted IL-1β activation in the absence of MLKL. However, when caspase-8 was inhibited genetically or chemically, the peroxisome (RIPK3–MLKL) could bind to the NLRP3 inflammasome to promote IL-1β activation, which is consistent with other previous studies demonstrating that both pathways are intracellular in origin. These findings indicate that RIPK3-induced inflammation may be driven by factors other than necroptosis and DAMP release [72, 73].

RIPK3 is a key regulator of inflammatory signaling, which can control various types of cell death pathways located downstream of death receptors and TLRs. Impairment of the RIPK3 signaling pathway is an important feature of many chronic inflammatory diseases and is related to the loss or excessive secretion of cytokines [74]. In rheumatoid arthritis (RA), IL-1β promotes mitochondrial membrane depolarization and exacerbates the acidosis-induced apoptosis of articular chondrocytes. IL-1β is produced by immune cells primarily through the activation of the NLRP3 inflammasome. In the absence of IAPs (inhibitors of the death protein family) and caspase-8, RIPK3 induces the production of the NLRP3 inflammasome, which induces the production of IL-1β. RIPK3 signaling plays an important role in the dysregulation of immune responses to arthritis induced by the transfer of K/BxN serum (serum from mouse models of severe arthritis) in mice. Therefore, joint inflammation and the inflow of polymorphonuclear (PMN) cells (white blood cells that promote inflammation) can be prevented by deleting RIPK3 in mice. RIPK3 deletion reversed joint dysregulation induced by caspase-8 deficiency under arthritic conditions and may have an inhibitory effect on arthritis induced by the transfer of K/BxN serum [75]. The levels of RIPK3 are remarkably high in the lung epithelial cells of patients with chronic obstructive pulmonary disease (COPD). Excess RIPK3 aggravates the disease and enhances inflammatory responses by regulating the activation of mitophagy [76]. In addition, RIPK3 is overexpressed in patients with nonalcoholic steatohepatitis (NASH) and is associated with liver inflammation and fibrosis [77]. Deletion of RIPK3 has been shown to alleviate CDAA-induced inflammation and fibrosis in mice [78]. RIPK3 may regulate peroxisome proliferator-activated receptor gamma (PPARγ), which has anti-proliferation activity. The increased expression of PPARγ in RIPK3-deficient mice has been shown to prevent tumor development. Therefore, targeting RIPK3 may represent a novel strategy for treating NASH and preventing disease progression. In pulmonary inflammation caused by viral infection, the loss of RIPK3 reduces the secretion of various inflammatory cytokines and chemokines, especially CXCL10 (also known as interferon-γ-inducible protein 10 [IP-10]) and chemokine ligand 2 (CCL2; monocyte chemoattractant protein 1 [MCP-1]). In addition, it can attenuate immune cell infiltration, especially neutrophil, macrophage, and T-cell infiltration, and alleviate lung injury [79, 80]. In cardiovascular diseases, RIPK3 acts as a key regulator of cell death and necrosis [81]. Abdominal aneurysm (AAA) is an aortic condition characterized by inflammation, loss of smooth muscle cells, extracellular matrix remodeling, and progressive aortic dilatation. Studies have validated that RIPK3 is overexpressed in AAA. In isolated aortic smooth muscle cells, knockdown or knockout of RIPK3 has been shown to attenuate the TNF-α-induced phosphorylation of p65 at Ser536 and expression of several pro-inflammatory cytokines. Phosphorylated p65 plays an important role in enhancing the transcription of NF-κB, which regulates cytokines, such as IL-6 and TNF, and VCAM-1. In addition, defects in RIPK3 can attenuate the formation of AAAs by inhibiting the necrosis and inflammation of aortic smooth muscle cells [82, 83].

### Role of RIPK3 in the cell cycle

RIPK3 regulates cell growth by controlling the cell cycle. Cell cycle progression is closely related to the direct or indirect phosphorylation of proteins associated with cell metabolism and development. RIPK3 can regulate changes in the structure of these proteins, thereby influencing cell growth [12]. RIPK3 deficiency can delay cell cycle progression and arrest cell division. For example, deletion of RIPK3 inhibits the generation of induced pluripotent stem cells (iPSCs). In RIPK3-deficient mouse fibroblasts (RIPK3-KO MEFs), the production of iPSCs can be induced by reducing the expression of genes that control cell cycle progression and cell division [84].

### Role of RIPK3 in autophagy

RIPK3 participates in etoposide-induced autophagy by phosphorylating UNC51-like kinase 1 (Ulk1) [12]. Functional complexes containing autophagy-associated (Atg) proteins drive the formation of autophagosomes, leading to canonical autophagy. Atg5-independent macroautophagy is called alternative autophagy [85]. Both Ulk1 and RIPK3 are involved in alternative autophagy. When stimulated by genotoxic stress, RIPK3 phosphorylates ULK1 at Ser746. Phosphorylated ULK1 is required for alternative autophagy [86]. Deletion of RIPK3 or inhibition of its kinase activity inhibits ULK1 phosphorylation and selective autophagy [87]. Therefore, RIPK3 can induce autophagy by phosphorylating U1K1.

### Role of RIPK3 in metabolism

RIPK3 not only affects inflammation but also plays an important role in mitochondrial metabolism. It may participate in glycolysis by regulating mitochondria-related metabolic enzymes [21]. Phosphorylation of RIPK3 activates metabolic enzymes involved in glycolysis, such as PYGL, GLUL, and GLUD1. These enzymes increase the levels of substrates for oxidative phosphorylation, which in turn promotes ROS production. Direct phosphorylation of PGAM5 by RIPK3 accelerates the dephosphorylation of DRP1 at S637 and activates its translocation to mitochondria, leading to mitochondrial fission and ROS production. Under TNF stimulation, phosphorylation of RIPK3 can activate the E3 subunit (PDC-E135) of the pyruvate dehydrogenase complex (PDC, also known as PDH), thereby promoting aerobic respiration and ROS production in cells. These findings suggest that RIPK3 regulates cell metabolism in addition to cell death [88].

## Diseases involving RIPK3

**Fig. 3:**
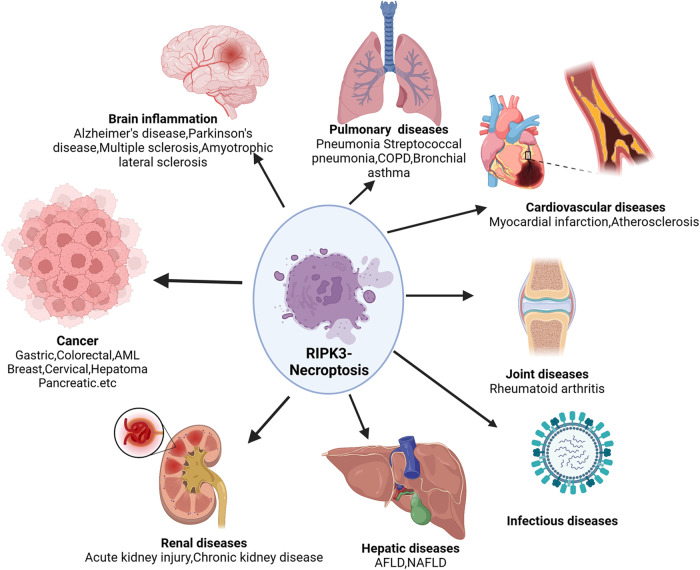
The role of RIPK3-mediated necroptosis in human diseases. Necroptosis is involved in the pathophysiological mechanisms of various clinical diseases, including infectious diseases, neurodegenerative diseases, hepatic diseases, pulmonary diseases, renal diseases, cardiovascular diseases, joint diseases, and cancer.

Necroptosis is a type of controlled cell death caused by disturbances in external or internal homeostasis and primarily relies on the kinase activity of MLKL, RIPK3, and (under some conditions) RIPK1 [89]. Because RIPK3 is essential for necroptosis, RIPK3-dependent cell death is a more accurate description of necroptosis [22]. RIPK3 has been shown to regulate and participate in necroptosis in a kinase-independent manner in vitro. RIPK3-dependent necroptosis has been associated with ischemic injury, inflammation, and neurodegenerative diseases [90] (Fig. 3). Therefore, RIPK3 is considered a potential target for preventing or treating these diseases. To date, numerous RIPK3 inhibitors have been identified for the treatment of various diseases.

### Ischemic injury

Various conditions can reduce blood perfusion in local tissues and organs, which causes ischemic damage to cells. Ischemic injury manifests as changes in membrane potential, cell swelling, and cytoskeleton disorder. In recent years, thrombolytic therapy, catheterization, arterial bypass surgery, organ transplantation, and other methods have been used to increase blood perfusion in ischemic tissues and organs, improving treatment outcomes in clinical settings. Ischemia–reperfusion injury (IRI), which refers to damage caused by the restoration of blood perfusion in ischemic tissues and organs, presents a different issue. IRI exacerbates dysfunction and structural damage, which frequently affects vital organs such as the heart, brain, kidney, and lung. As a cell death regulator, RIPK3 plays a crucial role in IRI.

In 2014, Luedde et al. demonstrated for the first time that RIPK3 expression was high in ischemic cardiomyocytes from mice with myocardial infarction (MI) [91]. The global knockout of RIPK3 has been shown to reduce infarct size and improve cardiac systolic function following acute IRI [92]. A study investigating the role of RIPK3 in cardiovascular failure in a model of MI with permanent coronary artery blockage showed that knockdown of RIPK3 attenuated unfavorable cardiac remodeling, dysfunctional cardiac hypertrophy, and inflammatory reactions after MI [93]. Notably, irrespective of RIPK1 and MLKL, RIPK3 activates Ca^2+^/calmodulin-dependent protein kinase IIδ (CaMK IIδ), resulting in myocardial necroptosis [94]. Because CaMK IIδ is an agonist of mPTP-mediated necrosis, mPTP opening and cardiomyocyte death can be prevented upon RIPK3 overexpression by pharmacologically inhibiting CaMK IIδ.

Atrial fibrillation, atherothrombosis, and embolism are the leading causes of ischemic stroke, resulting in severe disability including hemiplegia, speech impairment, impaired vision, and altered level of consciousness [95]. Using an animal model of middle cerebral artery occlusion (MCAO), Zhang et al. demonstrated that RIPK3 deficiency reduced necroptosis and neuroinflammation, protecting against ischemic brain injury [96]. To date, most studies have shown that necroptosis is the cause of a majority of negative effects of cerebral ischemia; therefore, targeting or inhibiting the downstream regulator RIPK3 represents a novel therapeutic strategy for ischemia. Dabrafenib, an inhibitor of serine/threonine kinase B-Raf V600E, is the only type I RIPK3 inhibitor approved for use in clinical trials [97]. It has been reported to decrease infarct size and attenuate the elevation of TNF-α levels to protect against ischemia-induced brain injury [22]. The most common cell-permeable RIPK3-selective kinase inhibitor GSK'872 [81] has been shown to reduce the size of lesions in mice with ischemic brain injury [98]. Li et al. found that the co-chaperone complex formed by HSP90 and CDC37 not only modulated the stability and functionality of RIPK3 and MLKL but also directly inhibited RIPK3. In addition, this complex was found to be involved in the activation of RIPK3 during necroptosis [99]. Kongensin A (KA), an HSP90 inhibitor, disrupts the association between HSP90 and CDC37. Compound 17AAG, another HSP90 inhibitor, disrupts the interaction between MLKL and RIPK3. Alvespimycin (17-DMAG) promotes the degradation of RIPK3 after inactivating HSP90, thereby preventing the formation of necrosomes, decreasing the phosphorylation of MLKL, and inhibiting TNF-induced necroptosis [22]. In future studies, direct or indirect inhibitors of RIPK3 should be used to gain important insights into the diagnosis and treatment of RIPK3-related diseases.

### Acute and chronic inflammation

Acute inflammation refers to localized redness, swelling, fever, or pain caused by trauma or bacterial infection. Chronic inflammation is primarily related to the destruction of immunological homeostasis and is mostly caused by the untimely and inadequate treatment of acute inflammation as well as by poor treatment outcomes, resulting in the gradual development of the disease. The treatment of incurable diseases may be improved by understanding the relationship between necroptosis and inflammation or immunological homeostasis. In 2011, Duprez et al. demonstrated that TNF-induced systemic inflammatory response syndrome (SIRS) was triggered by RIPK3-mediated necroptosis [100]. To date, studies on necroptosis have mostly focused on RIPK3, which has provided several novel therapeutic options.

Necroptosis plays an important role in hepatic cell death, which exacerbates chronic inflammation and fibrosis in the liver and eventually leads to cirrhosis and liver cancer. Patients with alcoholic fatty liver disease (AFLD) exhibit abnormally elevated RIPK3 expression in the liver, accompanied by other pathological abnormalities such as increased transaminase levels and lipid accumulation in liver cells [22]. Deletion of RIPK3, intraperitoneal injection of antisense oligonucleotides targeting RIPK3, and dabrafenib treatment have been shown to exert hepatoprotective effects in mouse models of acetaminophen toxicity, concanavalin hepatitis A, alcohol and diet intoxication, and NASH [94]. Although studies have established a relationship between hepatic RIPK3 levels and the severity of non-alcoholic fatty liver disease (NAFLD), the precise mechanisms through which RIPK3-dependent signaling contributes to the development of NAFLD remain unclear. A study showed that knockdown of RIPK3 significantly alleviated hepatic steatosis, liver injury, oxidative damage, fibrosis, and inflammation in mice with methionine-deficient and choline-deficient diet-induced NASH, whereas it aggravated hepatic steatosis and inflammation in mice fed a high-fat diet (HFD) [78].

RIPK3 affects various lung diseases, including asthma, idiopathic pulmonary fibrosis, acute respiratory distress syndrome, and bacterial and aseptic lung damage [21]. Huang et al. showed that RIPK3 expression increased in response to *Streptococcus pneumoniae* infection [101]. Necroptosis initiated by RIPK3 plays an indispensable role in host defense against *Streptococcus pneumoniae* infection, whereas RIPK3 deficiency decreases the bacterial clearance rate and aggravates lung inflammation and tissue damage, resulting in a high risk of mortality [101]. In neonatal mice exposed to urinary hydroxyproline (HYP), the RIPK3 inhibitor GW939B or genetic deletion of RIPK3 can decrease RIPK3 expression, which in turn alleviates lung inflammation and alveolar damage [102].

Necroptosis contributes to both acute and chronic kidney diseases and causes renal fibrosis [21, 103]. Martin-Sanchez et al. reported that RIPK3 deficiency inhibited NF-kB activation and alleviated renal inflammation in individuals with folate-induced AKI (FA-AKI) but did not prevent kidney failure. Bone marrow-derived cells that express RIPK3 are an essential early inflammatory factor in FA-AKI in chimeric mice [104]. Tumor necrosis factor-like weak inducer of apoptosis (TWEAK), a crucial pro-inflammatory cytokine that aggravates AKI and functions independently of necroptosis, causes renal inflammation mediated by RIPK3. Therefore, selective inhibition of myeloid-derived RIPK3 may represent an effective strategy for alleviating renal inflammation while preventing the risk of complications associated with systemic targeting of RIPK3 [104]. The abnormal accumulation of crystalline substances such as calcium oxalate (CaOx) in the kidney causes excessive renal tubular epithelial cell death, inflammation, and calcification. Hou et al. synthesized a novel RIPK3 inhibitor named complex-42 (Cpd-42), which reduces the accumulation of CaOx crystals in the kidney and attenuates their cytotoxic effects [97]. Notably, Cpd42 outperformed dabrafenib in terms of anti-necroptosis and anti-inflammatory activities.

Although necroptosis was not initially associated with TNF-induced acute intestinal injury, several recent studies have demonstrated that RIPK3 may significantly affect intestinal damage under some circumstances [105]. Welz et al. showed that RIPK3 had a protective effect on inflammation and cell death in Fas-associated protein with a novel death domain (FADD) deficiency-induced spontaneous colitis and ileitis models, indicating that RIPK3-mediated necrosis can lead to intestinal inflammation [106]. Additionally, RIPK3 inhibitors have been shown to reduce the necrosis of CD4 + T cells, differentiation of Th17 cells, and expression of pro-inflammatory cytokines [107], all of which are important colitis-inducing factors. These findings offer novel insights into the treatment of intestinal diseases.

RIPK3 has been associated with other autoimmune diseases, such as psoriasis, toxic epidermal necrolysis (TEN), RA, osteoarthritis (OA), Crohn’s disease, and pancreatitis [21]. According to earlier studies, RIPK3 protects mice against SIRS induced by TNF and sepsis caused by cecal ligation and puncture (CLP) [100]. However, recent studies have demonstrated that deficiency of RIPK3 or gasdermin D (GSDMD) can protect against TNF-induced SIRS, CLP-induced sepsis, or lipopolysaccharide (LPS)-induced septic shock. In addition, the positive feedback mechanism between the RIPK3/MLKL and GSDMD pathways and inflammation promotes the development of sepsis [108]. A study investigating the potential role of RIPK3 in the neuropathogenesis of herpes simplex encephalitis (HSE) showed that mice with RIPK3 knockout were more susceptible to HSE than wild-type mice after corneal inoculation with the herpes simplex virus (HSV), suggesting that RIPK3 delayed the progression of HSE. In addition, kinase-independent inflammation caused by RIPK3 and caspase-8 collectively inhibited viral replication in the brains of mice with HSE [109], providing a novel therapeutic strategy for HSE.

### Neurodegenerative diseases

Neurodegenerative disorders are defined by the deterioration of axons, the loss of myelin from neurons (brain and spinal cord cells), or both. RIPK1 and RIPK3 have been shown to form necrosomes, phosphorylate downstream MLKL, and activate MLKL-mediated necroptosis in multiple neurodegenerative diseases, including multiple sclerosis (MS), amyotrophic lateral sclerosis (ALS), Parkinson’s disease (PD), Gaucher’s disease (GD), and Alzheimer’s disease (AD) [21, 63].

The progressive demyelination of axons in the central nervous system is a hallmark of MS. In 2015, Ofengeim et al. reported increased TNF-α levels and aberrant caspase-8 activation in active white matter lesions in patients with MS [43]. In addition, increased levels of total and phosphorylated RIPK1, RIPK3, and MLKL have been observed in injured tissues [105]. From a therapeutic perspective, evidence mostly suggests that RIPK1 inhibitors (such as Nec-1) can protect oligodendrocytes; however, the role of RIPK3 in this process remains elusive and whether it may be safely silenced by small molecule inhibitors remains unclear. RIPK1 inhibition and RIPK3 deficiency have been reported to delay the onset of motor impairment and prevent axonal myelination abnormalities in mice with ALS expressing a mutant form of superoxide dismutase 1 (SOD1) (SOD1^G93A^ mice) [110]. A study showed that the protein expression of RIPK1, RIPK3, and MLKL was higher in postmortem substantia nigra samples from patients with PD than in control individuals, which is consistent with the pathological features observed in MS and ALS [111]. Notably, RIPK3 deficiency can significantly improve neurological and systemic sickness and enhance survival and motor coordination in mice with GD [19].

Furthermore, necroptosis is involved in other neurodegenerative diseases, such as spinal cord injury (SCI) and retinal degeneration [112, 113]. Reactive astrocytes and microglia express more RIPK3 and phosphorylated MLKL after SCI, and astrocytic death is significantly reduced in RIPK3 deficiency mice after SCI [94]. These findings highlight the relationship between RIPK3 and the improved maintenance of neurotrophic function.

In an animal model of retinal detachment (RD), the activator RIPK3 was recently discovered to increase more than 10-fold [114]. RIPK3 deficiency can effectively prevent necroptosis, alleviate oxidative stress, and reduce the release of apoptosis-inducing factors from mitochondria. In addition, it can significantly suppress cone cell death in mice with retinitis pigmentosa (RP) [115]. Therefore, RIPK3 is a viable therapeutic target for preventing and delaying the degeneration of photoreceptors in patients with RP.

## Role of RIPK3 in cancer

To overcome apoptosis resistance in anti-cancer treatment, researchers have paid more attention to the effects of necroptosis on cancer because it is a mode of RCD and can function when apoptosis is inhibited. As a key regulator of necroptosis, RIPK3 has been extensively investigated as well. However, existing studies suggest that RIPK3 has contradictory effects on cancer based on the type and specific development stage of cancer [116]. On the one hand, RIPK3 inhibits cancer development by directly killing cancer cells through necroptosis. Moreover, RIPK3 can induce anti-cancer immune surveillance mediated by T cells to limit tumor progression and limit tumor development by inducing and facilitating the secretion of cytokines and chemokines. On the other hand, RIPK3-mediated necroptosis creates an immunosuppressive tumor environment that promotes tumor growth, development, and metastasis [12]. In addition, RIPK3 can participate in tumorigenesis by (1) acting on various T cells to enhance cellular immunity; (2) inducing the aggregation of immunosuppressive myeloid cells, including myeloid-derived suppressor cells (MDSCs) and tumor-associated macrophages (TAMs), to promote immune evasion of cancer cells; (3) participating in PANoptosis.

### Inhibitory effects of RIPK3-mediated necroptosis on tumors

Studies have shown that tumor cell necroptosis frequently occurs in some solid tumors, including breast, liver, and lung cancers [117]. After a series of phosphorylation cascades, RIPK3-mediated necroptosis can inhibit tumor development through anti-tumor immunity. During this process, tumor cells are stimulated by TNF-α, TRAIL, or FasL as well as downstream TLRs, resulting in the activation of RIPK1. Additionally, under the effects of SMAC mimics (including SM164) and pan-caspase inhibitors (including Z-VAD-FMK), RIPK3 can interact with RIPK1 to form necrosomes, leading to the phosphorylation of MLKL and necroptosis of cancer cells [118, 119]. In addition, RIPK3 can promote the anti-tumor immune effects of CD8^+^ T cells by mediating necroptosis. DAMPs released by cancer cells undergoing necroptosis are recognized and bound by immature antigen-presenting cells (APCs), which eventually activate CD8^+^ T cells. Activated CD8^+^ T cells promote anti-tumor immunity via the perforin–granzyme or Fas–FasL pathway [120, 121]. This process occurs in various tumors, such as pancreatic adenocarcinoma (PAAD), colorectal cancer (CRC), and acute myeloid leukemia (AML) [122–124]. When the expression of RIPK3 is low, tumor cells exhibit resistance to necroptosis, resulting in tumor growth.

Snyder et al. showed that anti-tumor immunity can be initiated after necroptosis occurs in cells without tumor antigens in the tumor microenvironment (TME). In NIH-3T3 fibroblasts that have undergone necroptosis, activated RIPK3 promotes transcriptional responses mediated by the NF-κB pathway, increasing the levels of chemokines such as CCL3, CCL4, and CCL5 in TME. These chemokines enable the recruitment of APCs to TME, which internalize antigens and eventually activate the anti-tumor immune responses of CD8^+^ T cells. Notably, this process is limited to TME and the mechanisms through which RIPK3-mediated necroptosis of fibroblasts promotes antigen uptake and APC activation warrant further investigation [125].

### Promoting effects of RIPK3-mediated necroptosis on tumors

RIPK3-mediated necroptosis has been shown to promote tumor growth, development, and metastasis in some cancer models. Tumor cells can promote tumor progression by triggering immunogenic responses through necroptosis or enhancing the formation of an immunosuppressive TME. In several tumor cells, RIPK3 is often silenced by methylation near the transcriptional initiation site, which inhibits the activation of MLKL and fails to mediate necroptosis, consequently promoting tumor development [126].

In intestinal epithelial cells (IECs), RIPK3 is overexpressed when the mTOR signaling pathway is impaired. This process is often accompanied by the inhibition of Trim11-mediated autophagy-dependent degradation of RIPK3, which promotes the necroptosis of IECs and aggravates colitis, leading to the progression of inflammation-associated CRC [127].

Seifert et al. showed that RIPK3 was overexpressed and mediated necroptosis in pancreatic ductal adenocarcinoma (PDA) cells, inducing the release of the inflammatory factor SAP130 from tumor cells. Upon its recognition by the corresponding receptor Mincle, SAP130 induced the formation of an immunosuppressive TME, thereby promoting the progression of PDA [128].

Metastasis refers to the extravasation of tumor cells through endothelial cells. Therefore, the death of endothelial cells may accelerate tumor metastasis [129]. Upon activation of DR6, tumor cells can express the amyloid precursor protein to induce the necroptosis of endothelial cells, thereby accelerating tumor metastasis. On the contrary, specific treatment with RIPK3-deficient endothelial cells attenuates the death of endothelial cells, suggesting that RIPK3-mediated necroptosis can promote tumor metastasis [130].

Altogether, RIPK3-mediated necroptosis has significant effects on tumor growth, progression, and metastasis, indicating that targeting RIPK3-mediated necroptosis represents an effective strategy for inhibiting tumor development and progression in clinical practice. However, whether RIPK3 plays a pro- or anti-cancer role primarily depends on the relative quantity of the released chemokines and cytokines. Moreover, the mechanisms through which this balance is regulated warrant further investigation.

### Other roles of RIPK3 in tumors

As discussed in the abovementioned sections, RIPK3 can mediate the necroptosis of tumor cells or fibroblasts in TME. This process promotes the release of DAMPs from tumor cells or increased levels of chemokines such as CCL3, CCL4, and CCL5, which enhance antigen uptake and APC activation while promoting T cell-mediated anti-cancer immune surveillance. T cells not only participate in mediating anti-cancer processes but also lead to excessive damage to normal tissues. Therefore, the activities of T cells should be tightly regulated.

When the activity of caspase-8 is inhibited or lost, activated mature T cells undergo necroptosis. However, RIPK3 does not always act as a kinase in decreasing the abundance of some cells, such as mucosal-associated invariant T (MAIT) cells. Patton et al. found that in addition to mediating the necroptosis of MAIT cells, RIPK3 selectively inhibited the accumulation of MAIT cells in lymphoid organs as well as peripheral tissues. This inhibition was independent of the loss of caspase-8 or MLKL, indicating that downregulation of RIPK3 can increase the abundance of MAIT cells that are in a homeostatic state in the thymus, spleen, liver, and lung. However, RIPK3 did not affect the homeostasis and abundance of MAIT cells during infection with Francisella tularensis. Consequently, the mechanisms through which RIPK3 regulates the development of MAIT cells in the thymus warrant further investigation [131]. Notably, MAIT cells constitute 1–5% of innate T cells in the blood. In addition, they have high expression of the ABCB1 protein, which can promote drug efflux. Therefore, the anti-tumor effects of MAIT cells are not affected by chemotherapy [132]. Given that RIPK3 can be targeted to inhibit the accumulation of MAIT cells, targeting RIPK3 can be combined with chemotherapy for the treatment of cancer.

RIPK3 can assist cancer cells in evading immune surveillance by promoting the aggregation of MDSCs and TAMs. In CRC, downregulation of RIPK3 in MDSCs, which may be caused by factors from TME, eventually promotes the activation of the NF-κB pathway. Activated NF-κB promotes the transcription of COX-2, consequently catalyzing the production of prostaglandin E2 (PGE2). PGE2 downregulates RIPK3, promoting the immunosuppressive and carcinogenic activities of MDSCs [133]. MDSCs belong to colorectal cancer-infiltrating immune cells, which express Arg-1, iNOS/NOS2, and ROS and can induce immune tolerance by inhibiting the proliferation and activation of CD8^+^ T cells [134, 135]. In addition, they can differentiate into TAMs, promoting Th cells to inhibit tumor cells, and can secrete various cytokines, such as PGE2, to promote tumor cell proliferation [133]. Jayakumar et al. found that RIPK3 promoted the production of pathogenic Th17 cells by inducing the synthesis of IL17, IL23, and IL-1β in I-MDSCs and eventually accelerated the progression of intestinal tumors. However, the specific mechanism through which RIPK3 acts on I-MDSCs warrants further investigation [136].

In malignant melanoma and CRC, RIPK3 can interact with ZBP1 through RHIM, driving the recruitment of caspase-6 and caspase-8 to form the ZBP1–PANoptosome complex, which activates the NLRP3 inflammasome and induces PANoptosis. PANoptosis is an inflammatory RCD mechanism characterized by apoptosis, necroptosis, and pyroptosis, which can suppress tumor development [60, 137].

### Tumor treatment strategies targeting RIPK3

Although existing anti-tumor drugs can effectively induce apoptosis, the acquired resistance of cancer cells to chemotherapy and apoptosis are major challenges associated with the treatment of cancer in clinical practice. As a substitute for apoptosis, necroptosis can overcome this resistance, thereby improving the therapeutic efficacy.

Shikonin (SHI), a natural compound extracted from a Chinese herb, can mediate necroptosis to inhibit tumor growth by promoting RIPK3 phosphorylation, which subsequently promotes MLKL phosphorylation [138].

Additionally, prunetin (PRU), an O-methylated flavonoid, can be used to treat gastric cancer, as it can inhibit cancer cell proliferation through necroptosis by activating RIPK3 and phosphorylating MLKL [139].

Morgan et al. found that RIPK3 could be silenced by methylation in some cancer cell lines and primary cancer cells. Therefore, in analogous RIPK3-negative cancers, demethylating agents such as decitabine, RG108, and 5-azacytidine, which reduce DNA methylation near the transcription initiation site, can be used to restore the expression of RIPK3 [140]. Notably, the resistance of cancer cells to necroptosis is driven by certain oncogenes associated with the loss of RIPK3, such as BRAF and AXL. Therefore, inhibition of these genes may restore the expression of RIPK3. For example, Rizos et al. found that dabrafenib and vemurafenib effectively increased the expression of RIPK3 by inhibiting BRAF in patients with malignant melanoma [141]. In addition, Najafov et al. found that BMS-777607, which is an inhibitor of AXL/TYRO3, reactivated RIPK3 expression and restored necroptosis sensitivity in tumor cells [118]. Further investigation is warranted to understand the mechanisms through which these kinase inhibitors activate RIPK3 expression in tumor cells. In xenograft models, RIPK3 has been shown to not only reduce tumor growth but also increase the sensitivity of tumor cells to chemotherapy. Therefore, RIPK3 is a promising therapeutic target for tumors. However, novel methods should be developed to accurately regulate necroptosis in tumor cells.

RIPK3 can promote tumor growth in some RIPK3-positive or -overexpressing cancers. RIPK3 inhibitors can be used to suppress the occurrence and development of these cancers. Studies have shown that mTOR signaling relies on the gut microbiota. When RIPK3 overexpression is caused by the downregulation of the tumor suppressor gene TSC1 owing to an inappropriate diet, depletion of the gut microbiota induced by antibiotics or probiotics can attenuate the activation and expression of RIPK3. This process attenuates epithelial cell necrosis and alleviates colitis driven by excessive activation of mTOR [127].

RIPK3-mediated necroptosis is closely related to cisplatin, which is one of the most effective chemotherapeutic agents. On the one hand, cisplatin can inhibit the replication and synthesis of DNA by binding to it, thereby killing cancer cells. Notably, this process can lead to multiple side effects, especially nephrotoxicity. Studies have shown that necroptosis promotes cisplatin-induced nephrotoxicity, whereas inhibition of RIPK3 inhibits it [142]. On the other hand, cisplatin can trigger RIPK3-mediated necroptosis in tumor cells, leading to the release of cytosolic mtDNA, initiation of the cGAS–STING pathway, and secretion of IFN-I, consequently promoting the cross-activation of T cells through APCs. It is noteworthy that cisplatin enhances the abscopal effects of T cells in this process. Therefore, RIPK3 can be targeted to regulate the use of cisplatin in the treatment of tumors [143].

Researchers have developed other selective inhibitors targeting RIPK3. For example, GSK’872 can prevent the activation of RIPK3, thereby blocking the phosphorylation of the co-repressor TRIM28 at Ser473. This process regulates the transcription of TRIM28 during necroptosis and prevents TRIM28 from interacting with necrosomes, eventually inhibiting necroptosis [56]. GSK’840 and GSK’843 can inhibit RIPK3 by binding to its structural domain with high affinity [144]. However, the blockade of necroptosis by GSK'872 has been shown to accelerate the formation of a tumor-promoting microenvironment and exacerbate tumor progression in models of AOM-DSS-induced CRC [145]. In addition, HS-1371 competes with ATP to bind to the ATP-binding pockets of RIPK3, thereby suppressing the enzymatic activity of RIPK3 and inhibiting necroptosis. Only TNF-induced necroptosis is inhibited in this process, indicating that HS-1371 can specifically inhibit RIPK3-mediated necroptosis [146].

It is necessary to assess whether the abovementioned inhibitors can selectively inhibit certain cancer types. In addition, the synergistic effects of these inhibitors and immune checkpoint inhibitors should be evaluated in future studies.

To address the crosstalk between RIPK3-mediated necroptosis and other PCD mechanisms, researchers have proposed that RIPK3 can be delivered to tumor cells using engineered adeno-associated viruses (AAVs) to induce necroptosis precisely. This strategy has been successfully used to induce tumor-suppressive effects through necroptosis [125]. Given that many tumors undergo mutations, which complicate the disease, understanding the regulatory crosstalk between necroptosis and apoptosis is necessary.

## Conclusions

Necroptosis is an important cell death mechanism that is involved in various pathological conditions, including neurodegenerative diseases and cancer. Given that RIPK3 plays an important role in organ growth and tumor cell proliferation, it is considered a promising therapeutic target for various serious diseases. Understanding the modification and regulation of RIPK3 is important for understanding how cells survive or die through necroptosis via activation of NF-κB. The mechanisms underlying necroptosis and the role of RIPK3 in cell death-related signaling and disease pathogenesis warrant intensive investigation. In future studies, RIPK3 inhibitors should be used to gain valuable insights into the diagnosis and treatment of necroptosis-related diseases.
